# Handgrip strength, dynapenia, and health-related quality of life in older Korean adults

**DOI:** 10.1186/s12877-025-06218-8

**Published:** 2025-08-14

**Authors:** Keunjoong Yoo, Yong Soon Park, Hye Jin Kim

**Affiliations:** 1 Department of Family Medicine, Chuncheon Sacred Heart Hospital, Hallym University College of Medicine, 77 Sakju-ro, Chuncheon, 24253 Gangwon-do Republic of Korea; 2 Institute of New Frontier Research, Hallym University College of Medicine, Chuncheon, Gangwon-do Republic of Korea

**Keywords:** Health-related quality of life, Muscle strength, Aging, KNHANES

## Abstract

**Background:**

Handgrip strength (HGS) is a critical determinant of muscle function and has been strongly associated with various health outcomes, including health-related quality of life (HRQoL) in older adults. Although previous studies have explored the relationship between HGS and HRQoL, there is limited research on multiple muscle strength indicators in older Korean adults. In this study, we aimed to investigate the associations between multiple muscle strength indicators—maximal and relative HGS, dynapenia, and HGS asymmetry—and HRQoL in older Korean adults.

**Methods:**

In this cross-sectional study, we utilized data of 4,260 community-dwelling individuals aged ≥ 65 years who participated in the Korea National Health and Nutrition Examination Survey (KNHANES) 2016–2018. We assessed muscle strength using a digital dynamometer and defined dynapenia according to the criteria established by the Asian Working Group for Sarcopenia. HGS asymmetry was characterized by an interlimb difference of > 10%. HRQoL was evaluated using the EuroQol 5-Dimension (EQ-5D) index. Multivariable logistic regression models were employed to assess the associations between muscle strength indices and the high-risk group for low HRQoL (EQ-5D index ≤ 0.677), adjusting for potential confounders.

**Results:**

Of the 4,260 participants, 56.5% were female; 17.4% aged ≥ 80 years. A total of 8.3% of the participants were categorized as being at high risk for low HRQoL. Lower maximal HGS (adjusted odds ratio [aOR] = 1.065, 95% confidence interval [CI] = 1.023–1.108) and lower relative HGS (aOR = 1.136, 95% CI = 1.026–1.257) were significantly associated with poor HRQoL in women. Furthermore, dynapenia was independently correlated with a higher risk of impaired HRQoL in women (aOR = 1.497, 95% CI = 1.064–2.106), whereas no significant association was observed in men. HGS asymmetry was not significantly associated with HRQoL in either sex after adjusting for confounders.

**Conclusions:**

Reduced HGS and the presence of dynapenia were significantly associated with lower HRQoL, particularly among women. These findings highlight the critical role of muscle strength in maintaining the well-being of aging populations. Further longitudinal studies are warranted to validate these associations and elucidate potential causal mechanisms.

**Supplementary Information:**

The online version contains supplementary material available at 10.1186/s12877-025-06218-8.

## Background

Korea is undergoing rapid population aging, with the proportion of adults aged ≥ 65 years rising from 17.9% in 2022 to 18.4% in 2023, approaching the 20% threshold that defines a super-aged society [1, 2]. This demographic shift, driven by declining birth rates and increasing life expectancy, poses significant challenges to the nation’s economy, healthcare system, and social welfare infrastructure [3, 4]. One of the key challenges is maintaining health-related quality of life (HRQoL) in older adults, which is essential for independence, physical functioning, and overall well-being [5, 6]. At a societal level, improved HRQoL can reduce healthcare burdens and promote healthy aging [7, 8].

Among the various determinants of HRQoL, muscle strength is a critical factor. Studies have demonstrated that declining muscle strength is associated with greater disability, reduced mobility, and higher mortality risk [9, 10]. Conversely, individuals with higher muscle strength levels exhibit better physical performance and greater independence in daily activities, leading to enhanced HRQoL [11]. Furthermore, research suggests that muscle strength plays a more significant role compared with muscle mass in determining physical function and quality of life (QoL) in older adults, highlighting the importance for strength-based interventions [12]. This underscores the clinical significance of assessing and preserving muscle strength as a preventive strategy to maintain HRQoL and mitigate age-related functional decline.

Handgrip strength (HGS) is a widely used indicator of muscle strength, with both absolute and relative HGS gaining increasing attention in HRQoL research [13]. In this study, HGS was used as a proxy for overall muscle strength, as it reflects functional capacity and has been validated in older populations [12]. Additionally, HGS asymmetry is emerging as a potential marker for physical dysfunction and impaired QoL [14].

Despite the clinical relevance of these measures, few studies have comprehensively assessed the associations between diverse muscle strength indices and HRQoL in older adults. This study was conducted to fill a gap in the literature on the combined role of multiple strength indicators in HRQoL among older Korean adults. To address this gap, we aimed to investigate the relationships between HRQoL and multiple muscle strength indicators—including maximal HGS, relative HGS, dynapenia, and HGS asymmetry—in a nationally representative sample of older Korean adults. In particular, we performed sex-stratified analyses to explore potential sex-specific associations. Additionally, we sought to provide a comprehensive understanding about how these strength measures could be related to HRQoL in later life, with the goal of informing tailored strategies for supporting healthy aging.

## Methods

### Data source and study population

The present study utilized data from the seventh Korea National Health and Nutrition Examination Survey (KNHANES VII), conducted between 2016 and 2018. Since 1998, the KNHANES has been systematically administered to evaluate the health and nutritional status of the civilian, non-institutionalized Korean population. The KNHANES VII employed a cross-sectional, nationally representative design under the supervision of the Korea Centers for Disease Control and Prevention (KCDC; now Korea Disease Control and Prevention Agency [KDCA]). A stratified, multistage probability-cluster sampling approach was implemented to recruit 31,689 individuals across 13,248 households. Among them, 24,269 participants consented to participate in the survey, yielding a response rate of 76.5%.

**Fig. 1:**
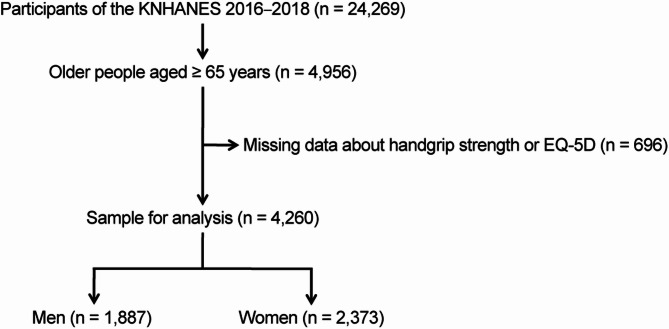
Flow diagram of participant selection from the Korea National Health and Nutrition Examination Survey, 2016–2018. Adults aged ≥ 65 years with complete data on handgrip strength and EQ-5D-3L were included in the final analytic sample (*n* = 4,260), which was stratified by sex

In this study, we included individuals aged ≥ 65 years from the KNHANES VII (*n* = 4,956). Participants with incomplete data on HGS and HRQoL (*n* = 696) were excluded following listwise deletion. According to the KNHANES protocol, individuals with conditions precluding valid grip strength measurement—such as upper limb amputation, paralysis, casts or splints, or recent hand/wrist surgery—were identified through pre-examination screening and excluded from testing. These participants were not included in the dataset and thereby excluded from the analytic sample. Given that missing values in these variables were frequently associated with missing data in other measures, their exclusion was deemed appropriate. Ultimately, data of 4,260 participants were retained for final analysis. The detailed selection process is illustrated in Fig. 1.

The KNHANES was conducted in full compliance with the Ethical Principles for Medical Research Involving Human Subjects. All participants provided written informed consent prior to participation, and all data were fully de-identified before public release. The study protocol was approved by the Institutional Review Board of Hallym University Chuncheon Sacred Heart Hospital (CHUNCHEON 2024-11-003).

### Assessment of HRQoL

The EuroQol 5-Dimension 3-Level (EQ-5D-3L) instrument and EQ-5D index scores were employed to evaluate HRQoL in older Korean adults. The EQ-5D-3L framework comprised five distinct health dimensions: mobility (MO), self-care (SC), usual activities (UA), pain/discomfort (PD), and anxiety/depression (AD) [15, 16]. Each dimension was rated on a three-level scale: no problems (= 1), some problems (= 2), and extreme problems (= 3). The participants were asked to report their current health status by selecting the statement that best described their condition in each of the five dimensions. This selection process generated a 1-digit score for each dimension, which could be combined into a 5-digit code representing the participant’s overall health state.

The EQ-5D index was derived by applying specific weighting factors to the five EQ-5D dimensions, generating a range of possible health states from 11111 to 33333. The index had a bounded scale from − 1 to + 1, with 1.0 representing the optimal health state, where all five dimensions indicated no problems (11111). In this study, EQ-5D index scores were computed utilizing predefined quality weights provided by the KCDC (now KDCA). The calculation formula was as follows: EQ-5D index = 1– (0.050 + 0.096×MO2 + 0.418×MO3 + 0.046×SC2 + 0.136×SC3 + 0.051×UA2 + 0.208×UA3 + 0.037×PD2 + 0.151×PD3 + 0.043×AD2 + 0.158×AD3 + 0.05×N3), where N3 represents the presence of extreme problems in any of the five health dimensions. The quality weights used in this formula were established through a population-based valuation study conducted by Lee et al. [17], which determined the preference weights for the Korean EQ-5D-3L based on a nationally representative sample. These validated weights were subsequently applied in this study to compute EQ-5D-3L index scores specific to the Korean population.

In our analysis, the EQ-5D index scores among the participants ranged between − 0.171 and 1.0, with a maximum value of 1.0 representing the highest possible health state. The distribution of EQ-5D index scores exhibited a left-skewed pattern, indicating a higher concentration of values near the upper end of the scale. The participants with EQ-5D index scores of 0.677 or lower were classified as being at a relatively high risk of deteriorated QoL. Although there was no universally established cutoff for the EQ-5D index, a threshold was necessary to effectively identify individuals in need of targeted health interventions. Rather than relying solely on statistical segmentation, we applied a functional classification approach. In the EQ-5D system, a person reporting no problems in any domain (11111) represented the best health state, while extreme problems in all domains (33333) represented the worst state. A response pattern of 22222, indicating “some problems” in all five dimensions, corresponded to an index score of 0.677. This value served as a meaningful threshold where functional impairment became evident, making it an appropriate criterion for identifying individuals requiring intervention. Although alternative methods such as percentile-based thresholds were considered, they might vary across populations and lack consistent clinical interpretation. In contrast, the “22222” response pattern corresponded to a moderate level of impairment across all five EQ-5D-3L domains and had been cited in prior literature as a functionally meaningful health state [15, 17]. Thus, we selected 0.677 as a clinically relevant threshold to functionally classify individuals at high risk, facilitating targeted health interventions.

### Muscle strength index

Muscle strength was measured using a digital grip strength dynamometer (TTK 5401; Takei Ltd., Tokyo, Japan). Before the assessment, trained examiners instructed the participants to remove any jewelry from their fingers or wrists to prevent interference. The examiners then provided a comprehensive explanation about the measurement procedure. The participants were positioned upright with their arms relaxed at their sides while holding the dynamometer. They were instructed to exert maximum grip force for 3 s, repeating the measurement three times per hand. To ensure accuracy, a minimum resting interval of 60 s was maintained between each trial.

Regarding analysis, the highest recorded grip strength value from the dominant hand was used. Relative HGS was computed by normalizing grip strength to the participant’s body mass index (BMI). In accordance with the Asian Working Group for Sarcopenia criteria, dynapenia was defined as an HGS below 28 kg in men and less than 18 kg in women [18]. Muscle mass was not measured in this study. Accordingly, dynapenia was defined based solely on low muscle strength, irrespective of muscle mass. This reflected clinically relevant muscle weakness that increased risks of impaired mobility and daily activity limitations, even without reduced muscle mass [10]. In this study, HGS asymmetry was determined based on a previously established threshold, defining asymmetry as a > 10% strength difference between hands [19, 20]. Participants exhibiting a strength difference exceeding this cutoff in either hand were classified as having HGS asymmetry. HGS asymmetry served as a surrogate marker for neuromuscular imbalance and might indicate early functional decline [14].

### Covariates

Sociodemographic variables analyzed in this study included age, sex, educational level, employment status, economic status, and marital status. Current health status was evaluated using the modified Charlson Comorbidity Index (CCI), along with additional health-related factors such as unmet medical needs, stress levels, hearing impairment, chewing difficulty, unintentional weight loss, BMI, waist circumference, and waist-to-height ratio. Lifestyle factors incorporated in the analysis comprised smoking status, alcohol consumption, participation in aerobic physical activity, and engagement in muscle-strengthening exercises. Detailed descriptions of variable measurements were obtained from the guidelines outlined on the KNHANES website (knhanes.kdca.go.kr/).

### Statistical analysis

Given the complex sampling design of the KNHANES, all statistical analyses were performed using complex sample analysis methods, accounting for sampling weights, stratification, and cluster sampling. Estimates were weighted according to the sampling rate, response rate, and distribution of age, sex, and regional proportions within the reference population. Continuous variables are summarized as means ± standard error (SE), while categorical variables are presented as weighted proportions (%) with SE. Differences in general characteristics between men and women were evaluated using the Student’s t-test for continuous variables and the Rao-Scott chi-square test for categorical variables. Associations between muscle strength indices and the high-risk group for HRQoL were analyzed separately according to sex. HGS was modeled as a continuous variable in all logistic regression analyses; estimated odds ratios (ORs) reflected the change in risk associated with each 1-kg lower value. Age-adjusted logistic regression models were employed to assess these associations, while multivariable logistic regression models were used to adjust for potential confounding factors. Statistical significance was defined as *P* < 0.05 for all two-sided tests. All analyses were performed using SPSS version 27 (IBM Corp., Armonk, NY, USA).

## Results

### General characteristics of the study population

Table 1 summarizes the general characteristics of the study population and sex-based comparisons. Of the 4,260 participants, 2,373 (56.5%) were female individuals; and 710 (17.4%) aged ≥ 80 years. A total of 8.3% of the participants were categorized as belonging to the high-risk HRQoL group, defined as having an EQ-5D index score of ≤ 0.677. A significant difference was observed in the prevalence of high-risk HRQoL between men (4.7%, 99 participants) and women (11.1%, 266 participants) (*P* < 0.001). The mean (SE) maximal HGS was 33.0 (0.1) kg in men and 19.4 (0.1) kg in women, while relative HGS was 1.41 (0.01) in men and 0.80 (0.01) in women. The prevalence of dynapenia was 23.3% in men and 38.2% in women, whereas HGS asymmetry was observed in 35.8% of men and 46.7% of women, both of which were significantly higher in women. There was no significant difference between sexes in household income (*P* = 0.775), modified CCI (*P* = 0.378), hearing impairment (*P* = 0.992), or unintentional weight loss (*P* = 0.752). However, most of the other variables were significantly different between men and women (all, *P* < 0.01).

**Table 1:** Sociodemographic, health-related, and lifestyle characteristics of the study population by sex

Characteristics	Total	(*n* = 4260)		Men	(*n* = 1887)		Women	(*n* = 2373)		*P*-value
[Sociodemographic factors]										
Age (years)										< 0.001
≥80	710	17.4	(0.8)	288	14.8	(1.0)	422	19.5	(1.1)	
75–79	987	23.7	(0.8)	432	20.7	(1.0)	555	26.0	(1.1)	
70–74	1166	25.5	(0.8)	532	27.2	(1.1)	634	24.2	(1.0)	
65–69	1397	33.4	(0.9)	635	37.3	(1.4)	762	30.3	(1.1)	
Education: ≤middle school	3080	72.4	(1.1)	1089	57.3	(1.4)	1991	83.9	(1.1)	< 0.001
Employment: no	2811	67.8	(1.0)	1091	58.6	(1.5)	1720	74.8	(1.1)	< 0.001
Household income										0.775
Lowest quintile	834	20.6	(0.8)	371	20.6	(1.1)	463	20.6	(0.9)	
Middle (Second–Fourth)	2514	58.0	(1.1)	1117	58.5	(1.4)	1397	57.6	(1.2)	
Highest quintile	889	21.4	(1.1)	388	20.9	(1.3)	501	21.8	(1.2)	
Spouse: no	1397	34.8	(1.1)	239	12.5	(0.9)	1158	52.1	(1.3)	< 0.001
[Current health status]										
Modified Charlson Comorbidity Index										0.378
≥2	375	8.5	(0.5)	191	9.3	(0.7)	184	7.9	(0.7)	
1	1048	24.5	(0.8)	467	24.5	(1.2)	581	24.5	(1.0)	
0	2837	67.0	(0.9)	1229	66.2	(1.3)	1608	67.6	(1.2)	
Unmet medical needs: yes	433	10.0	(0.6)	121	6.4	(0.7)	312	12.8	(0.8)	< 0.001
Stress: yes	794	18.7	(0.7)	235	12.5	(0.8)	559	23.5	(1.1)	< 0.001
Hearing impairment: yes	1360	31.6	(0.9)	622	31.6	(1.3)	738	31.5	(1.2)	0.992
Chewing difficulty: yes	1759	40.5	(1.0)	748	38.0	(1.3)	1011	42.5	(1.2)	0.008
Unintentional weight loss: ≥3 kg/year	604	14.7	(0.7)	273	14.4	(1.0)	331	14.9	(1.0)	0.752
Body mass index (kg/m^2^)		24.1	± 0.1		23.7	± 0.1		24.5	± 0.1	< 0.001
Waist circumference (cm)		85.5	± 0.2		86.7	± 0.2		84.3	± 0.2	< 0.001
Waist-to-height ratio, ×100		54.0	± 0.1		52.4	± 0.1		55.6	± 0.2	< 0.001
[Lifestyle factors]										
Smoking										< 0.001
Current smoker	386	9.0	(0.5)	338	18.1	(1.1)	48	2.0	(0.4)	
Ex-smoker	1237	28.5	(0.8)	1163	61.4	(1.3)	74	3.0	(0.4)	
Non-smoker	2612	62.5	(0.8)	377	20.5	(1.1)	2235	95.0	(0.6)	
Alcohol consumption: ≥2 days/week	707	16.9	(0.7)	596	32.9	(1.3)	111	4.6	(0.5)	< 0.001
Regular aerobic physical activity: no	2925	69.3	(0.9)	1200	63.4	(1.4)	1725	73.9	(1.1)	< 0.001
Muscle strengthening: ≤1 time/week	3513	82.2	(0.8)	1362	71.4	(1.2)	2151	90.5	(0.8)	< 0.001
[Muscle strength index]										
Maximum handgrip strength (kg)		26.2	± 0.1		33.0	± 0.2		19.4	± 0.1	< 0.001
Relative handgrip strength, ×10		11.0	± 0.1		14.1	± 0.1		8.0	± 0.1	< 0.001
Dynapenia: yes	1319	31.7	(1.1)	456	23.3	(1.2)	863	38.2	(1.6)	< 0.001
Handgrip strength asymmetry: yes	1812	42.0	(0.9)	689	35.8	(1.4)	1123	46.7	(1.2)	< 0.001
[Health-related QoL]										
5D-EQ index: high risk group	365	8.3	(0.5)	99	4.7	(0.5)	266	11.1	(0.8)	< 0.001

Data are presented as the estimated mean ± standard error or the unweighted frequency, estimated percentage (standard error), as appropriate. *P*-values are derived from the Student’s t-test for means or the Pearson chi-square test with the Rao-Scott correction using F statistic for proportions. High-risk group defined as EQ-5D index ≤ 0.677. EQ-5D, EuroQol 5-Dimension; QoL, quality of life

### Prevalence of high-risk HRQoL across different categories

Table 2 presents the estimated differences in the prevalence of various variables in the high-risk HRQoL group. Both men and women with dynapenia exhibited a significantly higher prevalence of high-risk HRQoL compared with those without dynapenia (all, *P* < 0.001). Regarding HGS asymmetry, a significantly high prevalence of high-risk HRQoL was observed only in women (*P* = 0.006). The prevalence of high-risk HRQoL was positively associated with advanced age, lower educational attainment, unemployment, higher modified CCI, unmet medical needs, stress, hearing impairment, chewing difficulty, and unintentional weight loss. Conversely, individuals who engaged in regular aerobic or muscle-strengthening exercises had a lower prevalence of high-risk HRQoL. Regardless of sex, both mean maximal HGS and relative HGS were significantly lower in the high-risk HRQoL group compared with the reference group (Table 3).

**Table 2:** Factors associated with high-risk group for health-related quality of life among older adults by sex

Characteristics	Categories	Men (*n* = 1887)		*P*-value	Women (*n* = 2373)		*P*-value
Age (years)	≥ 80	10.9	(2.1)	< 0.001	20.8	(2.4)	< 0.001
	75–79	7.8	(1.3)		10.8	(1.5)	
	70–74	2.6	(0.7)		9.3	(1.4)	
	65–69	2.0	(0.6)		6.7	(1.1)	
Education	≤Middle school	5.6	(0.8)	0.009	12.5	(0.9)	< 0.001
	≥High school	2.9	(0.6)		3.1	(1.1)	
Employment	No	6.2	(0.8)	< 0.001	11.9	(1.0)	0.013
	Yes	1.9	(0.5)		8.0	(1.2)	
Household income	Lowest quintile	6.1	(1.3)	0.334	17.2	(2.2)	< 0.001
	Middle (Second-Fourth)	4.7	(0.7)		10.6	(0.9)	
	Highest quintile	3.4	(1.2)		6.2	(1.2)	
Spouse	No	6.6	(2.1)	0.240	14.4	(1.2)	< 0.001
	Yes	4.4	(0.5)		7.7	(0.9)	
Modified Charlson Comorbidity Index	≥ 2	11.5	(2.6)	< 0.001	18.6	(3.0)	< 0.001
	1	5.0	(1.0)		13.8	(1.7)	
	0	3.6	(0.6)		9.3	(0.9)	
Unmet medical needs	Yes	11.8	(2.9)	< 0.001	21.6	(2.7)	< 0.001
	No	4.2	(0.5)		9.6	(0.8)	
Stress	Yes	11.6	(2.1)	< 0.001	23.0	(2.1)	< 0.001
	No	3.6	(0.5)		7.2	(0.8)	
Hearing impairment	Yes	7.2	(1.1)	< 0.001	17.2	(1.7)	< 0.001
	No	3.5	(0.6)		8.4	(0.8)	
Chewing difficulty	Yes	7.5	(1.1)	< 0.001	17.5	(1.4)	< 0.001
	No	2.9	(0.5)		6.0	(0.7)	
Unintentional weight loss	≥ 3 kg/year	7.8	(1.7)	0.012	16.8	(2.4)	< 0.001
	< 3 kg/year	4.1	(0.5)		9.5	(0.8)	
Smoking	Current smoker	4.2	(1.1)	0.834	20.3	(6.9)	0.140
	Ex-smoker	4.9	(0.7)		13.0	(4.0)	
	Non-smoker	4.3	(1.1)		10.6	(0.8)	
Alcohol consumption	≥ 2 days/week	2.4	(0.6)	0.001	6.7	(2.9)	0.219
	< 2 days/week	5.7	(0.7)		11.1	(0.8)	
Aerobic physical activity	None	5.8	(0.8)	0.001	13.3	(1.1)	< 0.001
	Regular	2.4	(0.6)		5.2	(1.0)	
Muscle strengthening	< 2 days/week	5.7	(0.7)	< 0.001	11.6	(0.9)	0.032
	≥ 2 days/week	1.7	(0.6)		6.4	(1.8)	
Dynapenia	Yes	10.0	(1.5)	< 0.001	16.5	(1.4)	< 0.001
	No	3.1	(0.5)		7.8	(0.9)	
Handgrip strength asymmetry	Yes	5.0	(0.8)	0.634	13.5	(1.3)	0.006
	No	4.5	(0.6)		9.0	(1.0)	

Data are expressed as the estimated percentage (standard error). *P*-values are derived from the Pearson chi-square test with the Rao-Scott correction using F statistic for proportions. High-risk group defined as EQ-5D index ≤ 0.677

**Table 3:** Comparison of muscle strength indices between high-risk and reference health-related quality of life groups by sex

Characteristics	Men					Women				
	High risk group		Reference group		*P*-value	High risk group		Reference group		*P*-value
Body mass index (kg/m^2^)	23.7	± 0.4	23.7	± 0.1	0.975	24.9	± 0.3	24.5	± 0.1	0.143
Waist circumference (cm)	87.9	± 1.2	86.7	± 0.2	0.334	85.5	± 0.7	84.1	± 0.2	0.066
Waist-to-height ratio, ×100	53.3	± 0.7	52.3	± 0.1	0.187	57.1	± 0.5	55.4	± 0.2	0.002
Maximum handgrip strength (kg)	28.8	± 0.9	33.2	± 0.2	< 0.001	17.1	± 0.3	19.7	± 0.1	< 0.001
Relative handgrip strength, ×10	12.2	± 0.4	14.2	± 0.1	< 0.001	7.1	± 0.2	8.1	± 0.1	< 0.001

Data are expressed as the estimated mean ± standard error. *P*-values are derived from the Student’s t-test for means

### Association between muscle strength indices and high-risk HRQoL

Table 4 presents the results of the logistic regression analyses examining the associations between muscle strength indices and the high-risk HRQoL group. Age-adjusted analyses revealed significant associations between all muscle strength indices and HRQoL, except for HGS asymmetry in older men. In the multivariable-adjusted model, the interaction terms between muscle strength indices and sex were not statistically significant (all, *P* ≥ 0.05). However, in sex-stratified analyses, significant associations were observed only in women, whereas no significant associations were found in men. Among older women, dynapenia was associated with a higher likelihood of belonging to the high-risk HRQoL group, with an adjusted OR of 1.497 (95% confidence interval [CI], 1.064–2.106). Although the adjusted OR for HGS asymmetry in older women was 1.392 (95% CI, 0.971–1.996), the association was not significant. Furthermore, in older women, each 1-kg lower HGS was associated with a 1.065-fold increase in the odds of high-risk HRQoL (95% CI, 1.023–1.108), while each 0.1-unit lower relative HGS was associated with a 1.136-fold increase (95% CI, 1.026–1.257). In contrast, no significant associations were observed between muscle strength indices and HRQoL in older men.

**Table 4:** Association between muscle strength indices and high-risk Korea National health and nutrition examination survey in older Korean adults stratified by sex

Characteristics	Men				Women			
	Model 1		Model 2		Model 1		Model 2	
Maximum handgrip strength (per 1-kg lower)	1.054	(1.005 − 1.104)	1.010	(0.954 − 1.069)	1.094	(1.055 − 1.135)	1.065	(1.023 − 1.108)
Relative handgrip strength, ×10 (per 1-unit lower)	1.154	(1.046 − 1.273)	1.091	(0.965 − 1.233)	1.222	(1.115 − 1.341)	1.136	(1.026 − 1.257)
Dynapenia (< 28/18 kg)	2.146	(1.221 − 3.772)	1.243	(0.649 − 2.380)	1.793	(1.305 − 2.465)	1.497	(1.064 − 2.106)
Handgrip strength asymmetry (> 10%)	1.027	(0.645 − 1.636)	0.848	(0.499 − 1.441)	1.456	(1.054 − 2.011)	1.392	(0.971 − 1.996)

Data are presented as adjusted odds ratio (95% confidence interval) derived from logistic regression. Odds ratios for continuous variables (e.g., maximum handgrip strength, relative handgrip strength ×10) reflect the change in risk per unit lower value (i.e., per 1-kg lower maximal HGS or per 1-unit lower scaled relative HGS). Model 1 was adjusted for age. Model 2 was adjusted for age, education attainment, employment status, household income (only women), marital status (only women), modified Charlson Comorbidity Index, unmet medical needs, stress, hearing impairment, chewing difficulty, unintentional weight loss, alcohol consumption (only men), aerobic physical activity, and muscle strengthening. High-risk group defined as EQ-5D index ≤ 0.677

## Discussion

We evaluated the associations between multiple muscle strength indicators and HRQoL in older Korean adults, offering critical insights into the influence of muscle strength on QoL in this population. Our findings revealed significant associations between maximum HGS, relative HGS, and dynapenia with reduced HRQoL, underscoring the importance for muscle strength as a determinant of well-being in older adults. Importantly, these associations exhibited notable sex-specific differences. Logistic regression analysis revealed that abnormalities in muscle strength indicators were significantly associated with an increased risk of low HRQoL in older women but not in older men. Specifically, older women with dynapenia exhibited an adjusted OR of 1.497 for belonging to the high-risk HRQoL group. Furthermore, each 1-kg lower maximal HGS and each 0.1-unit lower relative HGS were associated with 1.065-fold and 1.136-fold increases in the odds of high-risk HRQoL, respectively. Conversely, HGS asymmetry was not significantly associated with HRQoL.

The relationship between muscle strength and HRQoL in older adults has been extensively studied, with various HGS measures recognized as key indicators. Maximal HGS, a simple yet reliable measure of overall muscle strength, has consistently demonstrated positive associations with multiple health outcomes, including HRQoL. Previous studies have identified positive correlations between HGS and HRQoL and have established threshold values critical for maintaining optimal QoL [9, 21], findings that align with those in the present study. These results underscore the role of maximal HGS as a significant predictor of HRQoL in older adults. Collectively, the evidence highlights muscle strength as a critical determinant of HRQoL and emphasizes the potential benefits of interventions aimed to preserve or enhance muscle strength for promoting QoL in aging populations. Although previous studies have primarily examined absolute HGS in pooled or unstratified samples, our study incorporated multiple strength indicators—including relative HGS, dynapenia, and asymmetry—and applied a sex-stratified analytic framework. The associations with HRQoL were particularly pronounced in women, underscoring the need for sex-specific evaluation and targeted interventions.

The association between relative HGS and HRQoL in older adults has garnered increasing attention in recent years, with growing evidence supporting a strong and consistent positive relationship [22–24]. Relative HGS, which normalizes muscle strength to body size, offers a more precise representation of an individual’s physiological capacity and its implications for daily functioning and health outcomes [25, 26]. Furthermore, relative HGS has emerged as a superior predictor of all-cause mortality and metabolic risk in older adults, highlighting its clinical and public health relevance [26, 27]. Accounting for variations in body composition, relative HGS provides a more nuanced assessment of functional muscle strength than absolute measures such as maximal HGS, particularly in aging populations experiencing muscle mass reduction and fat mass accumulation. These findings emphasize the critical role of relative HGS in multiple dimensions of HRQoL. Interventions designed to preserve or enhance relative HGS might significantly contribute to improving the well-being of older populations. Additionally, relative HGS shows promise as a practical screening tool for identifying individuals at risk of HRQoL decline, thereby facilitating early intervention and targeted care.

Dynapenia has a significant impact on HRQoL in older adults, contributing to declines in physical and functional well-being, daily living activities, and overall QoL. Its effects are further exacerbated in conditions such as obesity and diabetes, where it accelerates physical QoL deterioration [28, 29]. Our findings revealed that dynapenia was significantly associated with lower HRQoL in women, likely reflecting its influence on physical function and independence. Among community-dwelling older adults, dynapenia is associated with lower physical performance, reinforcing the link between muscle weakness and functional impairment [30]. These findings underscore the pivotal role of dynapenia in physical functioning, a key determinant of HRQoL in aging populations. Interventions targeting the prevention or attenuation of dynapenia might help preserve physical function and improve QoL in older adults. Future research should prioritize the development of targeted strategies to mitigate dynapenia and evaluate its potential as a predictive marker for HRQoL decline.

We found no significant association between HGS asymmetry and HRQoL in older adults, contrary to prior research that reported associations between HGS asymmetry and adverse health outcomes [31, 32]. Several factors might account for this discrepancy. First, our definition of asymmetry as a difference of > 10% between hands, which aligns with previous studies [33], might have influenced the results. Alternative threshold values or continuous measures of asymmetry could yield different findings. Second, older adults might develop compensatory mechanisms to mitigate the adverse effects of HGS asymmetry on daily functioning and QoL. Lastly, the cross-sectional design of our study limits our ability to assess the long-term impact of HGS asymmetry on HRQoL. Although not statistically significant, asymmetry might still influence functional ability through impaired coordination or motor control, particularly in individuals with frailty. To assess the robustness of our asymmetry definition, we conducted additional sensitivity analyses using alternative thresholds (> 15%, > 20%) and a continuous variable. A significant association with high-risk HRQoL was found only in women at the > 20% cut-off. Moreover, asymmetry prevalence was higher among participants with dynapenia (particularly men) and those with EQ-5D ≤ 0.677 (particularly women) (tables not included). These findings suggest that while asymmetry might not consistently predict HRQoL across the population, it could reflect underlying physical vulnerability in specific subgroups. Correlation analysis with EQ-5D index was not performed due to methodological limitations in estimating valid coefficients from complex sample data. Longitudinal studies are warranted to determine whether the effects of asymmetry on QoL become more pronounced over time. Furthermore, it is plausible that older women might be more functionally affected by HGS asymmetry due to generally lower muscle reserves, a higher prevalence of dynapenia, and diminished hormonal protection after menopause. These biological differences might amplify the impact of asymmetry on physical performance and perceived QoL in women compared with men.

The observed sex-specific differences in the association between muscle strength indicators and HRQoL highlight the need for tailored approaches to muscle strength assessment and management in older adults. The stronger association between reduced muscle strength and lower HRQoL in older women suggests greater vulnerability to the physical and psychosocial consequences of muscle weakness [34]. Several factors likely contribute to this heightened susceptibility, including lower baseline muscle mass, hormonal differences, particularly the postmenopausal decline in estrogen, and greater age-related declines in muscle strength [10, 35, 36]. Additionally, the impact of reduced muscle strength on HRQoL in women may be exacerbated by socioeconomic and health-related factors [37].

In contrast, the weaker associations between muscle strength indicators and HRQoL observed in older men might reflect differences in the physiological and social determinants of well-being [38]. Biologically, older men tend to have higher baseline muscle strength and muscle mass, which might buffer the functional consequences of decline until more severe stages [10]. Behaviorally, men might underreport subjective impairments or perceive strength loss as less impactful on daily life. Methodologically, the lower prevalence of high-risk HRQoL in men might have limited the statistical power to detect associations. These factors might jointly explain the sex differences in strength–HRQoL relationships. In addition, higher baseline strength and a lower prevalence of dynapenia in men might act as protective factors. Greater employment participation and lower reported stress levels might also contribute to the maintenance of HRQoL despite the strength decline [37, 39]. These findings underscore the importance for sex-specific approaches to evaluating and supporting muscle function and perceived QoL. For instance, programs for older women may prioritize preserving or improving muscle strength through tailored resistance training, while interventions in men might focus on psychosocial and behavioral factors influencing QoL. Building on these observations, differentiated intervention strategies appear warranted. This sex-specific approach might enhance the effectiveness of public health interventions aimed to promote healthy aging and preserve functional independence in older adults.

This study has some limitations. First, its cross-sectional design precludes the ability to establish causal relationships between muscle strength indicators and HRQoL, making it susceptible to reverse causation. For instance, although low muscle strength might contribute to poor HRQoL, individuals with low HRQoL might also engage in reduced physical activity, leading to declines in muscle strength. Longitudinal studies are required to clarify these associations over time. Second, reliance on self-reported data in the KNHANES introduces potential biases, including recall bias and social desirability bias, which might have affected the accuracy of reported health and lifestyle factors. Additionally, participants with missing data on HGS and HRQoL were excluded from the analysis, potentially introducing selection bias. However, a comparison of baseline characteristics between excluded and included participants revealed no significant differences, suggesting minimal impact on the findings. Third, although HGS is a widely accepted indicator of muscle strength, it might not have fully captured the functional capacity of other muscle groups, particularly those in the lower body. Lastly, although we adjusted for key confounders, residual confounding might have persisted due to unmeasured factors, such as dietary intake, mental health status, and detailed socioeconomic indicators. Additionally, unreported comorbidities or genetic factors may have influenced the results. Addressing these limitations in future studies through comprehensive data collection, longitudinal designs, and advanced statistical methods would enhance the robustness and generalizability of the findings. Despite these limitations, this study has notable strengths. Utilizing KNHANES data ensured a nationally representative sample of older Korean adults, with analyses adjusted for the complex sampling design and sample weights, thereby improving generalizability. Furthermore, this study incorporated a comprehensive analysis of multiple muscle strength indicators, including maximal HGS, relative HGS, dynapenia, and HGS asymmetry. This multifaceted approach enabled a nuanced exploration of the relationship between muscle strength and HRQoL, providing a detailed understanding about how specific muscle strength deficits might impact QoL.

Additionally, classifying HRQoL levels with a focus on identifying high-risk groups in need of clinical intervention, the study enhances its practical applicability to developing targeted healthcare strategies. Nonetheless, we acknowledge that the absence of a universally accepted EQ-5D threshold might limit comparability across studies.

This study highlights muscle strength as a critical determinant of HRQoL in older adults, with notable sex-specific differences. Maximal HGS, relative HGS, and dynapenia were significantly associated with reduced HRQoL, particularly in women, underscoring the importance for muscle strength preservation. Routine muscle strength assessments could serve as practical tools for identifying at-risk individuals and guiding targeted interventions. Public health initiatives should prioritize resistance training, nutritional programs, and community-based strategies aimed at promoting physical activity and muscle strength to support healthy aging and mitigate healthcare burdens.

## Conclusions

We outline the significant association between muscle strength indicators and HRQoL in older Korean adults, with greater vulnerability observed in women. These findings support the integration of muscle strength assessments into routine health evaluations and the implementation of sex-sensitive interventions to address muscle weakness and its impact on HRQoL. Future research should investigate causal relationships through longitudinal studies and examine additional strength measures, such as lower limb strength, to provide a more comprehensive understanding about the role of muscle function in aging populations.

## Supplementary Information

Supplementary Material 1.

## Data Availability

The datasets generated and/or analysed during the current study are available from the KNHANES website: https://knhanes.kdca.go.kr/knhanes/main.do.

## Abbreviations

AD: anxiety/depression
BMI: body mass index
CCI: Charlson Comorbidity Index
CI: confidence interval
EQ-5D: EuroQol 5-Dimension
EQ-5D-3L: EuroQol 5-Dimension 3-Level
HGS: handgrip strength
HRQoL: health-related quality of life
KCDC: Korea Centers for Disease Control and Prevention
KDCA: Korea Disease Control and Prevention Agency
MO: mobility
OR: odds ratio
PD: pain/discomfort
QoL: quality of life
SC: self-care
KNHANES VII: seventh Korea National Health and Nutrition Examination Survey
SE: standard error
UA: usual activities
