# Supplementary material for: Handgrip strength, dynapenia, and health-related quality of life in older Korean adults
Source: BMC Geriatr. 2025 Aug 14;25:627. doi: 10.1186/s12877-025-06218-8 (PMC12355835; doi:10.1186/s12877-025-06218-8)
Supplement: Supplementary file 1 — Supplementary Material 1. [file 12877_2025_6218_MOESM1_ESM.docx]

Supplementary Table 1. Adjusted odds ratios for high-risk HRQoL according to handgrip strength asymmetry in older Korean adults stratified by sex

| Asymmetry variable | Men | | | Women | | |
| --- | --- | --- | --- | --- | --- | --- |
|  | aOR | (95% CI) | P-value | aOR | (95% CI) | P-value |
| HGS asymmetry >10% | 0.848 | (0.499−1.441) | 0.542 | 1.392 | (0.971−1.996) | 0.072 |
| HGS asymmetry >15% | 1.310 | (0.782−2.194) | 0.304 | 1.336 | (0.938−1.901) | 0.108 |
| HGS asymmetry >20% | 1.593 | (0.877−2.896) | 0.126 | 1.530 | (1.015−2.306) | 0.042 |
| HGS asymmetry (% difference) | 1.059 | (0.922−1.217) | 0.416 | 1.148 | (0.958−1.375) | 0.135 |

Data are presented as adjusted odds ratio (95% confidence interval) derived from logistic regression. Model was adjusted for age, education attainment, employment status, household income (only women), marital status (only women), modified Charlson Comorbidity Index, unmet medical needs, stress, hearing impairment, chewing difficulty, unintentional weight loss, alcohol consumption (only men), aerobic physical activity, and muscle strengthening. High-risk group defined as EQ-5D index ≤0.677. aOR, adjusted odds ratio; CI, confidence interval; HGS, handgrip strength; HRQoL, health-related quality of life.

Supplementary Table 2. Prevalence of handgrip strength asymmetry among older adults by HRQoL status stratified by sex

| Asymmetry variable | Men | | | | | Women | | | | |
| --- | --- | --- | --- | --- | --- | --- | --- | --- | --- | --- |
|  | EQ-5D ≤0.677 | | Reference | | P-value | EQ-5D ≤0.677 | | Reference | | P-value |
| HGS asymmetry >10% | 43 | (38.2%) | 646 | (35.7%) | 0.634 | 147 | (56.8%) | 976 | (45.4%) | 0.006 |
| HGS asymmetry >15% | 37 | (31.1%) | 375 | (20.7%) | 0.017 | 103 | (40.9%) | 631 | (29.7%) | 0.003 |
| HGS asymmetry >20% | 26 | (20.3%) | 203 | (11.1%) | 0.007 | 78 | (31.5%) | 404 | (19.2%) | <0.001 |

Data are expressed as the unweighted number (estimated percentage). Percentages represent column-wise proportions: the proportion of individuals with HGS asymmetry within each HRQoL group defined by EQ-5D index score (≤0.677 vs. >0.677). P-values are derived from complex sample chi-square tests. HGS asymmetry was defined as the absolute percentage difference between dominant and non-dominant handgrip strength. High-risk HRQoL was defined as EQ-5D index ≤0.677. HGS, handgrip strength; HRQoL, health-related quality of life.

Supplementary Table 3. Prevalence of HGS asymmetry among older adults by HRQoL status stratified by sex

| Asymmetry variable | Men | | | | | Women | | | | |
| --- | --- | --- | --- | --- | --- | --- | --- | --- | --- | --- |
|  | Dynapenia (+) | | Dynapenia (-) | | P-value | Dynapenia (+) | | Dynapenia (-) | | P-value |
| HGS asymmetry >10% | 191 | (42.1%) | 498 | (33.9%) | 0.005 | 442 | (49.9%) | 681 | (44.7%) | 0.054 |
| HGS asymmetry >15% | 132 | (29.0%) | 280 | (18.8%) | <0.001 | 295 | (33.7%) | 439 | (29.2%) | 0.068 |
| HGS asymmetry >20% | 84 | (17.7%) | 145 | (9.7%) | <0.001 | 211 | (24.5%) | 271 | (18.2%) | 0.003 |

Data are expressed as the unweighted number (estimated percentage). Percentages represent column-wise proportions: the proportion of individuals with HGS asymmetry within each dynapenia group (yes vs. no). P-values are derived from complex sample chi-square tests. HGS asymmetry was defined as the absolute percentage difference between dominant and non-dominant handgrip strength. Dynapenia was defined as HGS <28 kg for men and <18 kg for women. HGS, handgrip strength; HRQoL, health-related quality of life.
